# A review on the effect of nanocomposite scaffolds reinforced with magnetic nanoparticles in osteogenesis and healing of bone injuries

**DOI:** 10.1186/s13287-023-03426-0

**Published:** 2023-08-04

**Authors:** Hadi Sadeghzadeh, Hassan Dianat-Moghadam, Azizeh Rahmani Del Bakhshayesh, Daryush Mohammadnejad, Ahmad Mehdipour

**Affiliations:** 1 Department of Tissue Engineering, Faculty of Advanced Medical Sciences, Tabriz University of Medical Sciences, Tabriz, Iran; 2 Department of Genetics and Molecular Biology, School of Medicine, Isfahan University of Medical Sciences, Isfahan, Iran; 3 Department of Anatomical Sciences, School of Medicine, Tabriz University of Medical Sciences, Tabriz, Iran; 4 Student Research Committee, Tabriz University of Medical Science, Tabriz, Iran

**Keywords:** Nanocomposites, Magnetic nanoparticles, Scaffolds, Bone, Tissue engineering

## Abstract

Many problems related to disorders and defects of bone tissue caused by aging, diseases, and injuries have been solved by the multidisciplinary research field of regenerative medicine and tissue engineering. Numerous sciences, especially nanotechnology, along with tissue engineering, have greatly contributed to the repair and regeneration of tissues. Various studies have shown that the presence of magnetic nanoparticles (MNPs) in the structure of composite scaffolds increases their healing effect on bone defects. In addition, the induction of osteogenic differentiation of mesenchymal stem cells (MSCs) in the presence of these nanoparticles has been investigated and confirmed by various studies. Therefore, in the present article, the types of MNPs, their special properties, and their application in the healing of damaged bone tissue have been reviewed. Also, the molecular effects of MNPs on cell behavior, especially in osteogenesis, have been discussed. Finally, the present article includes the potential applications of MNP-containing nanocomposite scaffolds in bone lesions and injuries. In summary, this review article highlights nanocomposite scaffolds containing MNPs as a solution for treating bone defects in tissue engineering and regenerative medicine.

## Introduction

Bone tissue as a mineral connective tissue has important functions in the body, which is disturbed by many bone disorders such as bone fractures or defects caused by surgery, trauma, or primary tumor removal. Therefore, there is a growing need for safe and cost-effective treatment approaches for damaged bone tissues. On the other hand, the use of autograft and allograft products to repair damaged bone tissue is associated with the risk of disease transmission, chronic pain, infection, possible immunogenicity, lack of supply, and increased operation time [1]. It is anticipated that in the future, the high potential of bone for regeneration will lead to the introduction and development of bone tissue engineering (BTE) as a promising supporter of bone grafting techniques in regenerative medicine. Compared to conventional micro-sized materials, nanosized biomaterials have been shown to have the ability to induce cell adhesion and proliferation, and further bone regeneration [2]. Therefore, nanostructured biomaterials including biopolymer matrices and bioactive fillers in nanodimensions have been developed in nanocomposites-based scaffolds for use in tissue engineering, especially BTE [2–4]. Due to their small size and large surface area, these structures show good interaction with different cells and tissues and can facilitate absorption by cells.

In bone defects, in addition to the biological stimuli that originate from the understanding of bone biology, bone regeneration, and fracture repair, they can be stimulated by exogenous or endogenous physical factors such as fluid shear stresses, tensile and compressive stresses, and heat [5, 6]. Interestingly, magnetic stimulations from electromagnetic fields (EMFs) and static magnetic fields (SMFs) can also significantly progress bone repair and regeneration [7, 8]. Magnetic nanoparticles (MNPs), mainly superparamagnetic iron oxide nanoparticles (SPIONs), of small size (about 1–100 nm), with or without a magnetic field, can influence the function of ion channels in stem cells and regulate osteogenic differentiation. It also affects various biochemical pathways, scaffold activity, and growth factor turnover and can be used as a modulator in BTE [9–12]. In addition to dynamic magneto-mechanical stimulation, MNPs and magnetic fields provide the necessary growth factors, drugs, and gene transfections to accelerate the regeneration and repair of damaged bone [13–15]. Surprisingly, static magnetic field (SMF) expands the proliferation, migration, and differentiation of bone marrow mesenchymal stem cells (MSCs) into osteoblast-like cells and osteogenic cells [16]. In addition, electromagnetic field (EMF) also regulates the expression of type II collagen, thereby stimulating cartilage formation through the differentiation of human bone marrow MSCs into chondrocytes [17]. MNPs also have the potential to be used as magnetic resonance imaging (MRI) contrast agents for tracking implanted cells, bone regeneration, and scaffold degradation. Therefore, making an optimal magnetic scaffold in BTE can be very useful and effective.

MNPs are one of the most important nanomaterials that are assembled in nanocomposite scaffolds, and hence these types of scaffolds are called magnetic nanocomposite scaffolds. Among the scaffolds that support cell activity, nanofibrous scaffolds provide ideal substrates for cell adhesion and nutrient transport due to their highly porous structure, adjustable fiber diameter, and special network shape [18]. In particular, the integration of MNPs with polymer fibers solves the agglomeration problem and improves the stability of magnetic nanocomposite scaffolds produced with diverse morphologies [19, 20]. Compared to microfibers, nanofibers display better surface adsorption to proteins and enhanced cell adhesion and proliferation, which designates the importance of using a bimodal structure to achieve larger pores and an appropriate surface for cell adhesion and biological applications [21]. On the other hand, the use of these scaffolds in combination with MNPs increases cell attachment, and cell survival, and also increases the mechanical strength of the scaffold, all of which are needed for bone regeneration [22].

Therefore, it is important to have an overview of magnetic nanocomposite scaffolds used in bone tissue repair and regeneration. In this review, after the introduction of bone tissue engineering and types of magnetic nanoparticles and their application, the effects of various magnetic nanocomposite scaffolds used in bone tissue engineering on adhesion, penetration, proliferation, and specific differentiation of stem cells have been discussed.

## Bone tissue engineering

Bone tissue engineering (BTE) is an emerging field that aims to incorporate three components, including (1) osteogenic cells generating the bone tissue matrix, (2) a biocompatible scaffold mimicking bone extracellular matrix (ECM), and (3) physico-chemical stimuli affecting cell behavior. A successfully engineered bone product has no permanent graft site complications, exhibits adequate vascularization, and does not induce an immunologic reaction at the defect site [23].

Over time, the polymer scaffold that supports the tissue regeneration process is absorbed or replaced by newly produced bone tissue [24]. As three-dimensional structures with high porosity, various scaffolds are widely used in bone tissue engineering and support cell-biomaterial interactions, cell adhesion, growth, and migration [2, 4]. At the same time, they also facilitate the transport, survival, proliferation, and differentiation of progenitor cells [25]. A suitable biomaterial for the construction of scaffolds in bone tissue engineering should have critical behaviors such as osteo-induction and osteo-conduction and also be able to maintain bone integrity so that the components of the biomaterial integrate into the surrounding bone tissue [26, 27]. In addition, easy sterilizability, easy fabrication, non-thrombogenicity, and stability in different chemical and mechanical conditions are other key parameters that should be considered in biomaterials used in BTE [28]. Furthermore, when the scaffolds are implanted in the bone defect, they must have sufficient stability and elasticity to withstand the suture site and, in addition, be able to support bone formation with homogeneous morphology. Finally, the implanted scaffold must be degraded in a controlled manner in vivo without or with a minimal degree of inflammatory or toxic side effects [29, 30].

## Magnetic nanoparticles and magnetic nanocomposite

According to the number of nanophases (i.e., < 100 nm) of homogeneous solid materials used to make nanocomposites, they can be one-dimensional (e.g., thin films and surface coatings), two-dimensional (e.g., nanowires or nanotubes), or three-dimensional (e.g., multilayer structures). In other definitions, in the nanocomposite structure, the interphase spacing is repeated in the nanoscale range [31, 32]. Nanoparticles (NPs) are materials that are articulated with all three external dimensions at the nanoscale, exhibit a high total interfacial area and high surface-to-volume ratio, and create extraordinary interactions with molecular and supramolecular structures in biological environments [33]. Magnetism is the magnetic moment per unit volume of a particle, which is typically dependent on the spin or orbital energy that the dipole possesses. Therefore, the magnetic behavior is affected by the sample temperature and the degree of magnetic order [34].

In general, the synthesis of magnetic nanoparticles is done using two processes: top down or bottom up, which include different techniques such as ultrasonication, radiation, electrochemical, vapor deposition, and microwave [35, 36]. In this regard, MNPs (metal oxide NPs and SPIONs) can be fabricated using various methods, including co-precipitation, surfactant-assisted techniques, solgel and hydro-thermal processing, and emulsion techniques. These methods are used to control the structure, surface morphology, and stability of the manufactured nanoparticles [37–39]. Among the challenges of creating monodisperse magnetic nanostructures are size control, particle surface effects, and dipole interactions. However, some new chemical synthesis methods have made it easier to make functional MNPs. MNPs with a size of less than 30 nm are the dominant superparamagnetic nanoparticles [40, 41]. For example, superparamagnetic magnetite nanocrystal clusters (SMNC) as developed MNPs can be synthesized via mini-emulsion/solgel and polyol systems and used for multifunctional applications including combined drug targeting and cell imaging [42].

Iron oxide nanoparticles in two forms, Fe_3_O_4_ and Fe_2_O_3_, are the most common MNPs that are usually synthesized by conventional co-precipitation method [43] and are widely used in MRI applications for imaging cancer cells, and in vivo tracking and monitoring of cells and transplanted tissues [44]. The excellent biocompatibility and low toxicity of these types of MNPs led researchers to use them in the biomedical field, especially for monitoring engineered tissues [45]. Fe_3_O_4_ has been accepted by the US Food and Drug Administration (FDA) for clinical use [46]. Potential applications of MNPs include organ regeneration, tissue implants, drug delivery, imaging, and improved diagnostics.

In line with what was mentioned, in the field of tissue repair and tissue engineering, the unique feature of large surface area in biodegradable nanofillers complements the critical parameters mentioned above for classical scaffolds (e.g., biocompatibility, physicochemical stability, support of cell adhesion, and differentiation) which ultimately effectively improves the BTE approach [47, 48]. In bone tissue engineering, various technologies, including the foam replica method [49], solvent casting and particulate-leaching [50], freeze-drying [51], phase separation [52], gas foaming [53], rapid prototyping [54], and electrospinning [55], have been used to develop and introduce efficient nanocomposite scaffolds with controllable size and porosity that show a high surface-to-volume ratio. However, most scaffolds cannot be controlled after implantation in vivo, and since the repair process can only be finalized by scaffolds in vivo, then the repair will not always be good enough. Therefore, one of the suggested approaches to achieve appropriate tissue repair and the possibility of controlling tissue fate using external stimuli is the construction of composite scaffolds containing magnetic nanoparticles.

Since MNPs can respond to EMF, scaffolds containing MNPs can also respond to external magnetic fields. In addition, MNPs and magnetic responsive scaffolds (MRS) can deliver various peptide agents, and also improve implant stabilization [56], improve mechanical properties and biocompatibility [57, 58], and improve the wettability of the scaffolds. Moreover, MNPs and MRS increase alkaline phosphatase (ALP) activity and osteogenic gene expression of bone cells [59, 60]. Therefore, combining the potentials of MNPs and nanocomposite scaffolds leads to the development of implantable and functional magnetic nanocomposites (MNCs) [61, 62]. The most important aspect of these scaffolds is the magnetic response, which leads to great progress in tissue engineering such as magnetic patterning of cells and 3D tissue-like structures [63–65].

Recently, Panseri et al. made magnetic scaffolds composed of hydroxyapatite/collagen and MNP and showed that the presence of magnetic particles causes the attraction of growth factors and cells [65]. Also, embedding iron oxide nanoparticles in macro-porous ferrogel scaffolds led to the production of MNC with a porous structure optimized for cell delivery [66]. In addition, doping of magnetic poly (1-caprolactone)/iron into hydroxyapatite leads to the fabrication of MNCs capable of simultaneous use in the repair of damaged tissues and the treatment of further hyperthermia [67]. It is noteworthy that each particle in the structure of MNPs owns a single magnetic domain, and thus, the incorporation of MNPs into scaffolds generates a nanoscale magnetic field that affects the cells and scaffolds interaction in the exposed microenvironments. In addition, the endogenous force exerted by MNPs or exogenous stimulation using a magnetic field has been shown to affect multiple cell surface receptors as well as associated signaling pathways to modulate cell function toward a specific target [68–70].

Magnetic field stimulation also accelerates the bone healing process by enhancing the integration of scaffolds and host bone and increases calcium content for bone density and new bone formation [71–73]. It has also been shown that functionalized MNPs injected near the scaffold can be absorbed in the damaged site under the influence of an external magnetic field to promote tissue regeneration [74]. Remarkably, as previously reported for wound healing, a moderate external static magnetic field can modulate osteoblast differentiation even without the presence of MNPs [75–77]. In general, the intrinsic magnetic properties of MNCs or exposure to far magnetic fields combined with appropriate mechanical support lead to the regulation of signaling pathways and various biological responses, the promotion of osteogenic cell differentiation, and ultimately bone regeneration and injury repair [78, 79].

Despite all the positive features and wide applications, one of the limitations of using magnetic nanoparticles is their instability and low solubility in water environments [80]. To deal with this problem and to increase their stability, using a hydrophilic polymer substrate and covering magnetic nanoparticles is recommended [81]. Also, to prevent aggregation, magnetic nanoparticles should either be stabilized by electrostatic or steric repulsion [82]. Another limitation of MNPs is the toxicity related to size, shape, and chemistry, which should be considered before clinical use. Therefore, while MNPs seem to have significant therapeutic, restorative, and diagnostic potential, long-term evaluation is necessary to reduce human health risks [83]. Hence, they require comprehensive pre-evaluation in terms of bio-distribution and biocompatibility. It should also be noted that MNPs, either alone or in a polymer substrate, must be quickly removed from the body after reaching the therapeutic endpoint. Considering all these, it is concluded that the development of MNPs for therapeutic purposes is in its infancy. It appears that the collaborative multidisciplinary science that will deliver the next generation of MNPs is related to theranostics where combined diagnosis and treatment will eventually become part of standard medical practice [84]. Therefore, it is expected that in the near future interdisciplinary research in the fields of biology, engineering, physics, and chemistry can provide new technologies based on magnetic nanoparticles to improve global health.

## Molecular effect of MNPs on osteogenic differentiation and bone repair

The differentiation of mesenchymal stem cells (MSCs) plays a crucial role in bone repair and regeneration. The process of osteogenic differentiation of MSCs is a complex and intricate process that involves the activation of several signaling pathways and transcription factors, such as Wnt/β-catenin, Notch, BMP/TGF-β, PI3K/Akt/mTOR, MAPK, PDGF, IGF, FGF, and Ca2+ pathways [85–87]. Magnetic stimulations from MNPs alter cell behavior and have great potential for BTE applications. The use of magnetic field stimulation has been shown to activate multiple sensitive receptors on the surface of cells and trigger related signaling pathways, resulting in enhanced cell activity [69]. Continuous application of magnetic field acts as sustained stimulation to further increase cellular activity in the bone defect [70]. Additionally, magnetic field stimulation can cause better integration of scaffolds and host bone, promote new bone density through increased calcium content, and ultimately accelerate bone healing. These nanoparticles that have a particle size smaller than 30 nm exhibit a superparamagnetic effect and can be seen as individual magnetic domains [40, 41]. Therefore, it is reasonable to assume that MNPs in the microenvironments within the scaffolds can create a nanoscale magnetic field and exert a micromagnetic driving force at the interface between the scaffolds and cells, thereby affecting the fate of cells and subsequently in tissue repair. This happens by activating several sensitive receptors on cell surfaces, increasing cell activity and enhancing bone formation during bone healing.

**Fig. 1:**
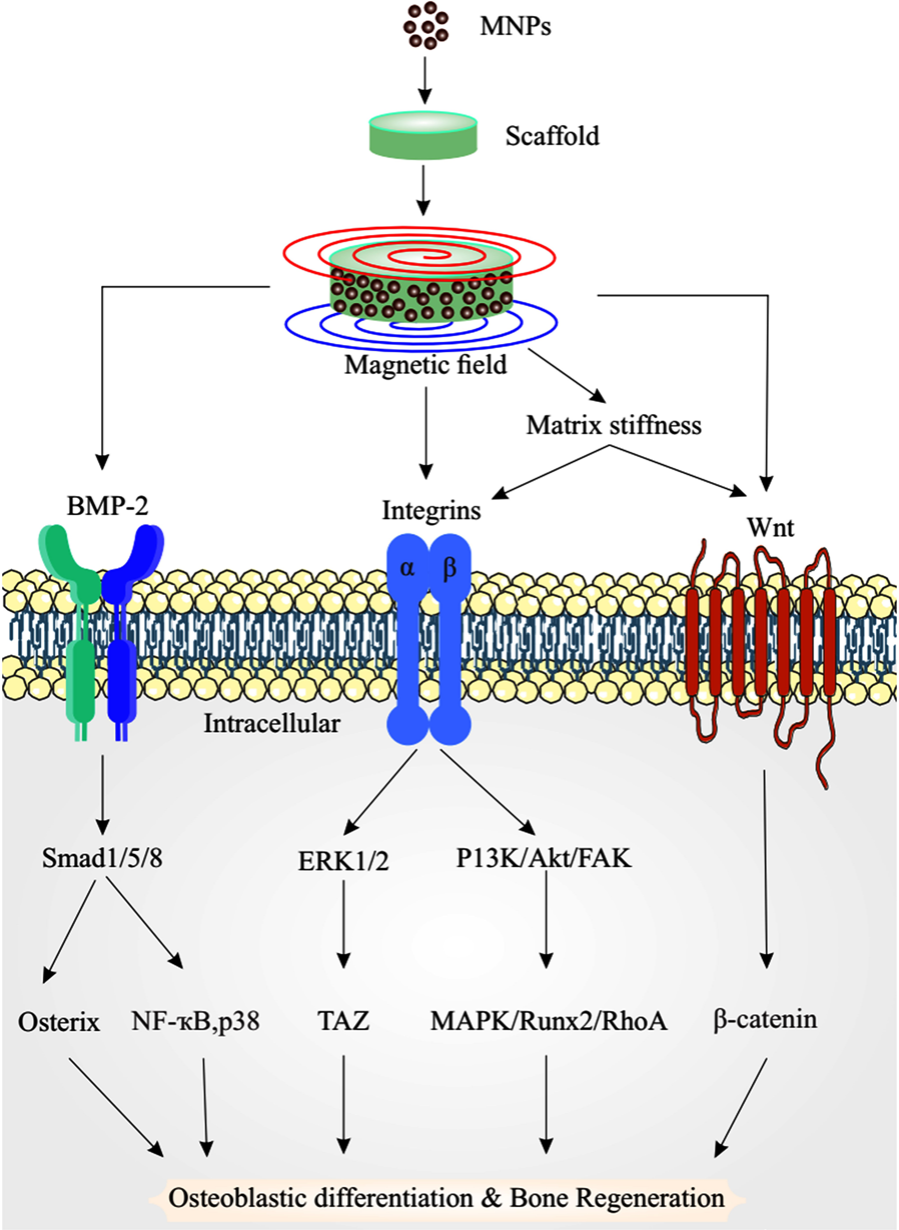
Schematic image of the signaling pathways activated by magnetic nanocomposite scaffolds inducing osteogenesis (Designed by CorelDRAW 2019)

The incorporation of MNPs into the structure of scaffolds can promote the osteogenic differentiation of MSCs in several ways. MNPs increase the surface roughness of scaffolds, which enhances surface energy and protein absorption, promoting the interaction between cells and scaffolds and activating the integrin signaling pathway. From the perspective of molecular mechanisms, magnetic induction stimulates cellular receptors and ECM components to activate the signaling pathways including the Wnt/β-catenin, integrin, and BMP2 (Fig. 1). The studies indicated that exposure of stem cells to a low-frequency magnetic field enhanced the expression of Wnt3a. This ligand activates the Wnt/β-catenin pathway and improves osteogenic differentiation of human bone marrow-derived mesenchymal stem cells (BMSCs) [88, 89].

ECM proteins (*e.g.*, Integrins and collagens) offer a cell adhesion substrate for cellular functions that allows the development of subsequent mineralization. Integrins are transmembrane receptors that can potentially transmit ECM physicochemical and mechanical conditions to the cells. MNPs have excellent specific hardness and strength as well as large specific surface area. These properties can be utilized to enhance the mechanical properties of scaffolds through their ability to reinforce and strengthen the structure [90]. The mechanical properties of scaffolds, including their stiffness, can influence the fate of combined stem cells. High matrix stiffness can induce MSCs to differentiate into osteoblasts, so that the upper and lower stiffness of the matrix causes the osteoblast and nerve differentiation of MSCs, respectively [91]. Therefore, Integrins can mediate the matrix stiffness affecting cells and regulate osteogenic differentiation of stem cells. From a molecular point of view (Fig. 1), in the presence of high matrix stiffness, integrins can activate osteogenic differentiation-related signaling pathways such as the PI3K/Akt pathway and FAK or ERK1/2 phosphoric acid toward enabling stem cells to differentiate into osteoblasts [92, 93]. Indeed, matrix stiffness regulates the ERK pathway, which subsequently up-regulates the expression of osteogenic genes [94]. At the same time, integrin α5 activation upregulates the expression of collagen type I and Runx2 (runt-related transcription factor 2), leading to increased deposition of calcium [95]. Notably, signaling pathways such as BMP and Wnt are critical pathways promoting osteogenic differentiation that activated by integrin β3 and phosphorylation of GSK3 (glycogen synthase kinase-3) via integrin-linked kinase and β-catenin nuclear translocation [96]. The BMP signaling pathways mediated by Smad1/5/8 phosphorylation [97].

In a study using decellularized cancellous bone scaffolds coated with various proportions of collagen/HA mixtures, MSCs implanted on the high-stiffness scaffolds demonstrated superior osteogenic ability [98]. Moreover, matrix stiffness can affect cell morphology, which is related to the cytoskeleton and intercellular interactions [99]. Cells on stiffer matrices are more spread out, with enhanced expression of vinculin, promoting the formation of focal adhesion and activation of β-catenin signaling, and inducing bone formation and remodeling [100]. For example, the treatment of rat BM-MSCs via graphene oxide (GO) combination with Fe_3_O_4_ (MGO), leads to the up-regulation of BMP2 and Wnt/β-catenin signal pathways. It also provides cell protective activity where ferrous iron from Fe_3_O_4_ reacts with hydrogen peroxide (H_2_O_2_) to produce hydroxide and hydroxyl radicals [101].

Regarding osteogenic differentiation, bioinformatics analyses of genes microarray test revealed that MNPs induced MSCs osteogenic differentiation through modulation of expression of genes (e.g., ALP, COL1, RUNX2, and OCN) and induction of the signal pathway such as MAPK (classic mitogen-activated protein kinase) [11]. ZEB2 is a regulatory factor that inhibits the BMP/Smad-related osteogenic differentiation. SPIONs support sustained ossification by upregulating the INZEB_2_ (long non-coding RNA) in MSCs, that overexpress INZEB_2_ and downregulate ZEB_2_ expression [102]. In this regard, it was observed that gelatin sponges containing SPION increased bone regeneration with about a 1.5 times increase in BMD and BV/TV (bone volume per tissue volume) in comparison with gelatin sponges SPION-free [12].

Similar to the magnetic field, a pulsed electromagnetic field (PEMF) also induces and activates ERK1/2 and PKA (protein kinase A) signaling pathways to promote bone repair [103]. PEMF also increases the Ca^2+^ concentration in the cytoplasm by opening ion channels (voltage-gated) and inducing the expression of osteogenesis-related genes [104]. Moreover, static magnetic field (SMF) has a similar effect and induces the expression of Wnt and Smad4 and other signaling pathways related to osteogenic differentiation, such as nuclear factor-κB, p38, and JNK (c-Jun N-terminal kinase)/MAPK pathways [101, 105]. For example, SMFs (15 mT) and PCL/MNPs composites synergistically upregulated the osteogenesis-related genes such as Runx2 and Osterix and induced alkaline phosphatase activity in mice osteoblasts to enhance osteoblastic differentiation [78]. Overall, these pathways detect changes in microenvironments, cytoskeletons, cell membranes, and nucleoproteins induced by magnetic forces [106, 107]. In summary, the incorporation of MNPs into scaffolds by creating a magnetic field can influence their surface roughness, wettability, and mechanical properties, increase their surface energy, protein absorption, cell-scaffold interaction and stiffness, and subsequently regulate bone differentiation.

On the other hand, angiogenesis supports the delivery of nutrients and signaling factors in damaged tissues to promote the formation of new tissues [108, 109]. In this case, magnetic systems affect the secretory function of osteoblasts and indirectly on other cells. The significant point in angiogenic responses is that magnetism upregulates VEGF (vascular endothelial growth factor) and angiogenin-1 genes in endothelial cells and promotes the formation of capillary tubes [110, 111].

## Magnetic nanocomposite scaffolds in bone tissue engineering

Along with other medical applications (i.e., drug delivery, biosensors, imaging, etc.), nanomedicine has a special place in regenerative medicine. Today, nanocomposite biomaterials have emerged as a new class of biocompatible materials that are used as bioactive, absorbable, and nanosized fillers for developing matrix structures [48]. Various studies have been conducted to prepare efficient magnetic nanocomposite scaffolds for use in BTE **(**Table 1**)**.

**Table 1:** Applied MNPs in bone tissue engineering

MNP composition	Scaffold material	Osteogenic impact	Study model	Advantage (s)	Disadvantage (s) or limitation	Refs
Fe_3_O_4_	Nanohydroxyapatite (nHAP) + chitosan + collagen organic matrix	MNPs improve cell adhesion, proliferation, and osteogenic differentiation	In vitro/In vivo model of SD rats’ skull defects	MNPs induced the infiltration of osteoblasts into the center of the defect	Uncontrolled magnetocaloric effect of MNPs in Earth’s magnetic field	[58]
FeCl_3_/FeCl_2_	Mag Gel: Type II collagen (Col II), hyaluronic acid (HA), and PEG	Mag Gel promotes BMSCs proliferation, differentiation, and viability	In vitro study on BMSCs	-Long-term of cytocompatibility - Decreased the rate of degradation and improved the integrity of the scaffold for a long time due to the cross-linking of the gels	Requirement of an external magnet to determine the combined effects of magnetic field and magnetic scaffold on cell function	[17]
Fe_3_O_4_/γ-Fe_2_O_3_	Poly-ε-caprolactone (PCL)	Significant increase in MSC differentiation and ALP activity	In vitro study on MSCs	Longer stability, biocompatibility, and optimal surface chemistry for MSCs adhesion were observed	The actual inducer of the molecular effects is unknown	[112]
Iron-doped	PCL/hydroxyapatite	Showed a high impact on BMSC growth and adhesion	In vitro/ in vivo study on rabbit animal model	Rapid prototyping process, the potential to develop a 3D construct for the reconstruction of extensive diaphyseal bone defects	Limited vascularization and poor tissue functionality	[113]
Gd^3+^	Multifunctional nHAp/PCL	Induced early osteogenic differentiation of h-MSCs	In vitro study on MSCs	Provide MRI imaging	The need for real-time in vivo imaging	[114]
IONPs	PLGA/PCL + Au NPs	γ-Fe_2_O_3_ NPs significantly enhance the osteogenic differentiation of ADSC	In vitro study on ADSCs	Increased hydrophilicity and elasticity & Introduced a magneto-sensing protein called iron—sulfur cluster assembly protein 1 (ISCA1)	The proliferation of ADSCs showed non-significant variations among the different scaffolds	[115]
Fe_3_O_4_	PLLA/PGA	Magnetic fields enhance the adhesion, migration, and differentiation of osteocytes	In vitro study on MG63 cells*/ *In vivo study on rabbit radius bone defect	Magnetic scaffolds induced significant blood vessel tissue formation	Their weak magnetic properties caused a low rate of bone regeneration	[116]
Fe_3_O_4_	Mesoporous bioactive glass (MBG)/PCL	MNPs stimulate cell proliferation and ALP activity	In vitro study on h-BMSCs	MNPs effectively induced ECM mineralization and osteogenesis-related gene expression in h-BMSCs	MNPs did not influence scaffolds to induce apatite mineralization	[117]
Magnetite	Calcium phosphate cements (CPCs)	Magnetite improves cell adhesion, spreading, proliferation, and increased ALP expression in BMSCs	In vitro study on h-BMSCs	The incorporation of ultrafine MNPs dramatically changed the properties of CPCs	Irreproducibility of the exact percentage of MNPs	[118]
IONP	CPCs	IONP increases ALP activity and osteogenic gene expressions	In vitro study on human dental pulp stem cells (h-DPSCs)	IONPs improved the CPC wetting, more protein adsorption, and greater cell attachment and spreading	Magnetism did not give significant influence here without an EMF & animal studies are also needed	[59]
γ-IONP	CPC	IONP-CPC induces more active osteogenesis than CPC control	In vitro study on h-DPSCs/ In vivo study on rat mandible defects	No adverse tissue reaction was observed. γ-IONP-CPC generated new bone that was four folds greater than that of CPC control	Variation in microstructure affects results, and different cells may respond differently to SMF	[119]
SrFe_12_O_19_	Mesoporous bio glass/chitosan porous scaffold (MBCS)	MNPs activates BMP2/Smad/Runx2 pathway	In vitro study on h-BMSCs/ In vivo study on tumor-bearing rat	MBCS indicates excellent bone regeneration and photothermal therapy for treating the tumor-related bone defects	Due to the heterogeneity of the tumor microenvironment, the results may not be reproducible	[120]

In 2014, Wang et al. used the electrospinning technique to create a composite material containing of MNPs based on Fe_3_O_4_. They used poly (l-lactic acid) (PLLA) polymer concentrations of 2% and 5% to load nanoparticles and finally showed that the spreading and attachment of fibroblast cells to the fabricated nanocomposites was well done [67]. In addition to unique magnetic properties and high surface area, Fe_3_O_4_ MNPs have high specific hardness and specific strength, which improves the mechanical attributes of magnetic scaffolds [121]. Due to the advantages of Fe_3_O_4_ MNPs, several studies have used biomaterials containing Fe_3_O_4_ MNPs for BTE. Pan et al., who used the extrusion process to integrate Fe_3_O_4_ into Poly-l-lactide polymer to make Fe_3_O_4_/Poly-l-lactide composites, finally showed the ability of osteogenic differentiation without a cytotoxic effect on fibroblast cells in vitro [122].

Similarly, D’Amora et al. prepared the poly(e-caprolactone)/iron-doped hydroxyapatite scaffolds that enhanced cell proliferation in vitro while causing minimal toxicity [123]. Also, in a study regarding the induction of bone formation by MNPs, the incorporation of SPIONs (superparamagnetic iron oxide nanoparticles) (Fe_2_O_3_) into calcium phosphate cement (CPC) led to the construction of nanocomposite scaffolds. It was reported that compared to CPC scaffolds without SPIONs, the shape of the scaffold surface caused better adhesion and osteogenic differentiation of human dental pulp stem cells (hDPSCs). These scaffolds also released SPION into hDPSCs, thereby regulating osteogenic gene expression and ALP activity, and bone matrix mineral synthesis in cells [59, 124].

Another study using ultrasonic irradiation o synthesize nanocomposite scaffolds containing bacterial cellulose (BC), Fe_3_O_4_, and hydroxyapatite (HA) NPs indicated that HA and Fe_3_O_4_ NPs were uniformly distributed on the surface and cross-section of the BC matrix. They have also shown high porosity (81.1%) and good mechanical attributes (9.87 MPa and 1.85 GPa). BC/Fe_3_O_4_/HA developed MNC showed significant attachment to living bone cells without toxicity and also induced cell differentiation and proliferation [125]. With all these advantages, preparing magnetic nanofibers with a high content of Fe_3_O_4_ NPs is difficult. To solve this problem, the combination of different manufacturing methods, such as the combination of cooperative assembly methods with electrospinning technology, is used. This strategy has effectively overcome the mentioned challenge and displayed excellent magnetic performance, BMSCs biocompatibility, and viability, and more importantly, provided a dynamic cell culture microenvironment for a model of actual 3D growth under alternating magnetic field without direct contact with cells [126].

**Fig. 2:**
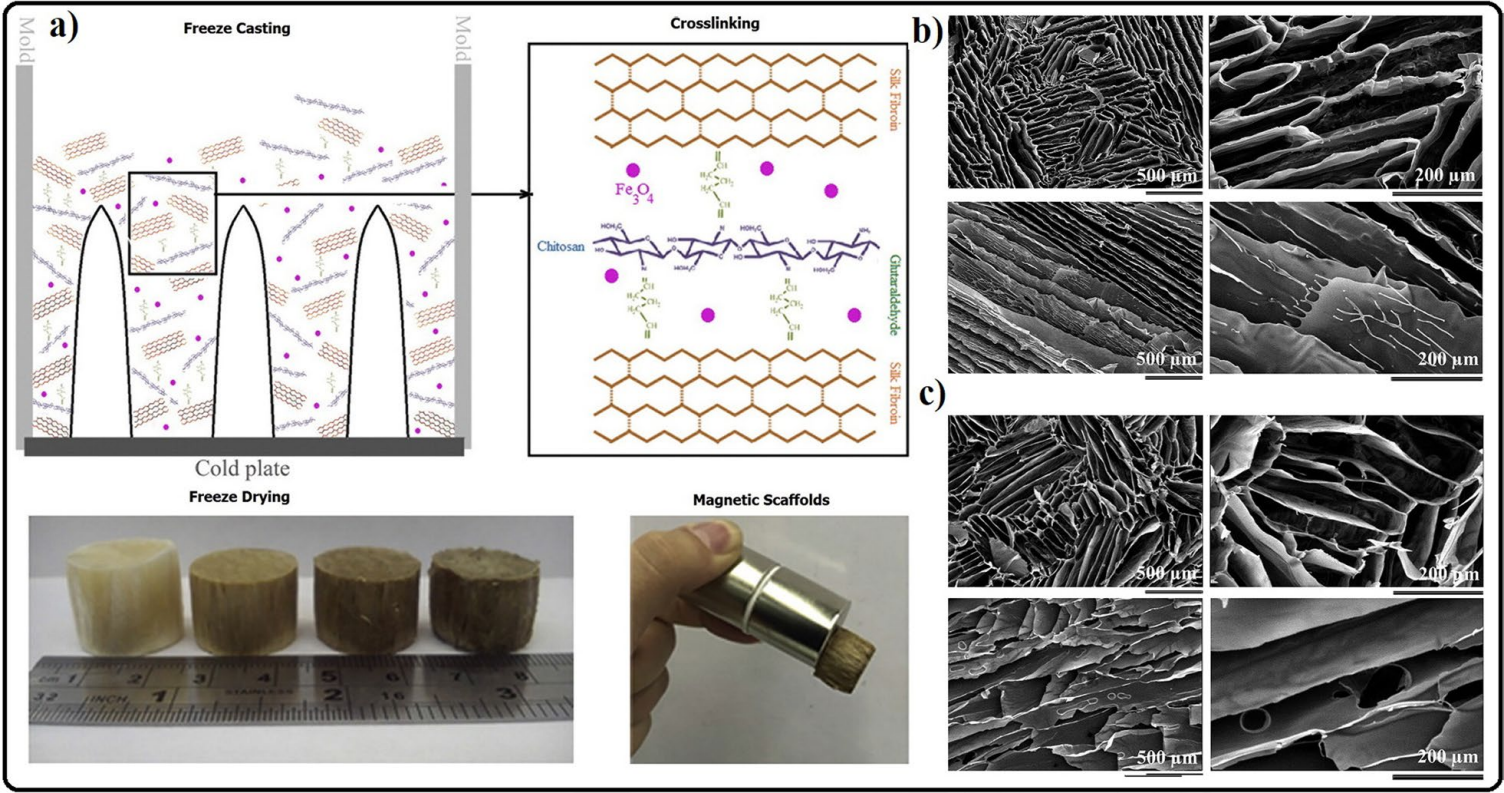
**a** Schematic illustration of chitosan/silk/MNPs scaffolds preparation by freeze-casting method. **b** SEM images of chitosan/silk and **c** chitosan/silk/MNPs. Reprinted from Ref. [127]. Aliramaji, S., A. Zamanian, and M. Mozafari, Super-paramagnetic responsive silk fibroin/chitosan/magnetite scaffolds with tunable pore structures for bone tissue engineering applications. Materials Science and Engineering: C, 2017. 70: p. 736–744, Copyright (2023), with permission from Elsevier

The freeze-casting method is another strategy to gain highly interconnected porous magnetic scaffolds appropriate for BTE. For example, a study investigated the effect of different percentages of MNPs integrated into the chitosan/silk fibroin structures. As shown in the SEM micrographs in Fig. 2, the structure of the scaffolds was layered. It was also shown that the incorporated nanoparticles did not meaningfully affect the microstructural features of the scaffolds. However, interestingly, the samples containing 1% MNPs showed a lower degradation rate. In this regard, MNPs-free scaffolds showed the highest degradation, which was attributed to the hydrophobic nature of MNPs in scaffold structures. In addition, in the mentioned study, it was shown that a higher percentage of MNPs decreases cell viability, which indicates the need to optimize the percentage of MNPs used in BTE [127]. MNPs can be considered a single nanoscale magnetic domain that may influence cell membrane ion channels to control cellular responses [128, 129]. Even though the magnetic field intensity of the nanoscale particle is very low, the overall effect is likely to be enhanced by increasing the MNP amount, thus having a more substantial impact on cellular responses. These somehow create a dynamic environment that influences the surrounding cellular reactions.

Cobalt-based NPs are another type of MNPs that has a high magnetic saturation. On the other hand, due to the high biocompatibility of Fe_3_O_4_, their combined properties with cobalt in the form of CoFe_2_O_4_ MNPs are considered as one of the most useful spinel ferrites, which have suitable characteristics such as large magneto-crystalline anisotropy, medium saturation magnetization, mechanical stiffness, high inductance, high magnetic resistance, and chemical stability [112, 130]. Cobalt can also be used in combination with zinc, as in a study where cobalt-zinc ferrite (CZF) NPs were fabricated and incorporated up to 3 wt% into poly(ε-caprolactone) nanofiber composites scaffolds. In the resulting nanofiber, the diameter was reported to decrease to 466 nm. Also, hydrophilicity, mechanical stability, biodegradability, and biocompatibility were demonstrated in the presence of EMF [131].

Similar to this study, in other studies, the integration of MNPs into other biological materials, such as ceramic or calcium phosphate scaffolds, has been used to induce cell proliferation, differentiation, and bone formation in vivo. For example, in various studies, adding HA nanoparticles to composite scaffolds or calcium phosphate scaffolds significantly increased the attachment, proliferation, and differentiation of osteoblast cells under EMF and showed tissue biocompatibility [132].

The cell-ECM and cell–cell interactions regulation, predicting drug sensitivities, providing nutrients or stimuli, and removing waste products are essential conditions for the native 3D-microenvironment of bone that classical 2D plastic scaffolds in single-cell cultures cannot provide. As a result of the absence of these conditions, cell death or loss of function and impaired repair occurs [133, 134]. These limitations are overcome by a novel 3D scaffold that provides a high degree of porosity, allowing cell interaction, waste removal, and oxygen/nutrient diffusion. In addition, 3D scaffolds show a more natural morphology of cells compared to 2D structures and provide better differentiation of them into physiologically relevant tissue. It should be mentioned that the size of the pattern, and the topography of the scaffold and the cell type can determine morphology of cell, migration, proliferation, and differentiation [135]. These observations indicate the importance of the fabrication of proper microstructures and designing 3D biomimetic scaffolds that mimic the native tissue. Of course, the design of such microenvironments needs the use of computational modeling and computer-aided instruments for tissue engineering [136].

To develop magnetic nanocomposite scaffolds containing Fe_3_O_4_, a 3D printing technique can be used and scaffolds with uniform pore size, unique morphology, and architecture can be prepared. For example, in a study, this technology was used to make magnetic nanocomposite scaffolds containing Fe_3_O_4_, bioactive glass, and polycaprolactone (Fe_3_O_4_/MBG/PCL). The results showed that these scaffolds increased proliferation rate and ALP activity and induced the human bone marrow mesenchymal stem cells (h-BMSCs) differentiation toward bone repair and regeneration [117]. Another study used PCL/Fe/HA nanoparticles to prepare 3D fully biodegradable magnetic nanocomposite scaffolds. The in vitro results showed a 2.2-fold increase in its potential in the growth of BMSCs. In addition, in the mentioned study, after four weeks, the magnetized scaffolds were filled with new bone in vivo, which confirmed the excellent tissue compatibility of the magnetic scaffolds [113]. In another study, electrospinning followed by the layer-by-layer assembly (LbL) was used to assemble a film of SPION on the surface of a magnetic scaffold containing PCL/PLGA. The results showed that hydrophilicity, elasticity, and surface roughness were greatly increased, which subsequently significantly increased cell attachment and osteogenic differentiation of ADSCs [115].

Notably, implantable scaffolds with larger pores provide a bone-like micro-environments that increase cell proliferation and migration [137, 138]. Moreover, structures with high porosity allow the fast diffusion of medium, oxygen, and metabolites, creating a favorable micro-environment for cells [139]. Addressing these notes, the solvent casting technique alongside the overlap of nylon template structures is another way to develop bioinspired 3D porous nanocomposites scaffolds comprised of piezoelectric polymers, such as PVDF (polyvinylidene fluoride) and CoFe_2_O_4_. Studies on these scaffolds show that their structures are alike to the spongy bone (pore sizes 5–20 µm). Also, due to the natural crystallization process with magneto-mechanical and magneto-electric stimulation, larger pores with more interconnectivity are created after removing the nylon template [140].

**Fig. 3:**
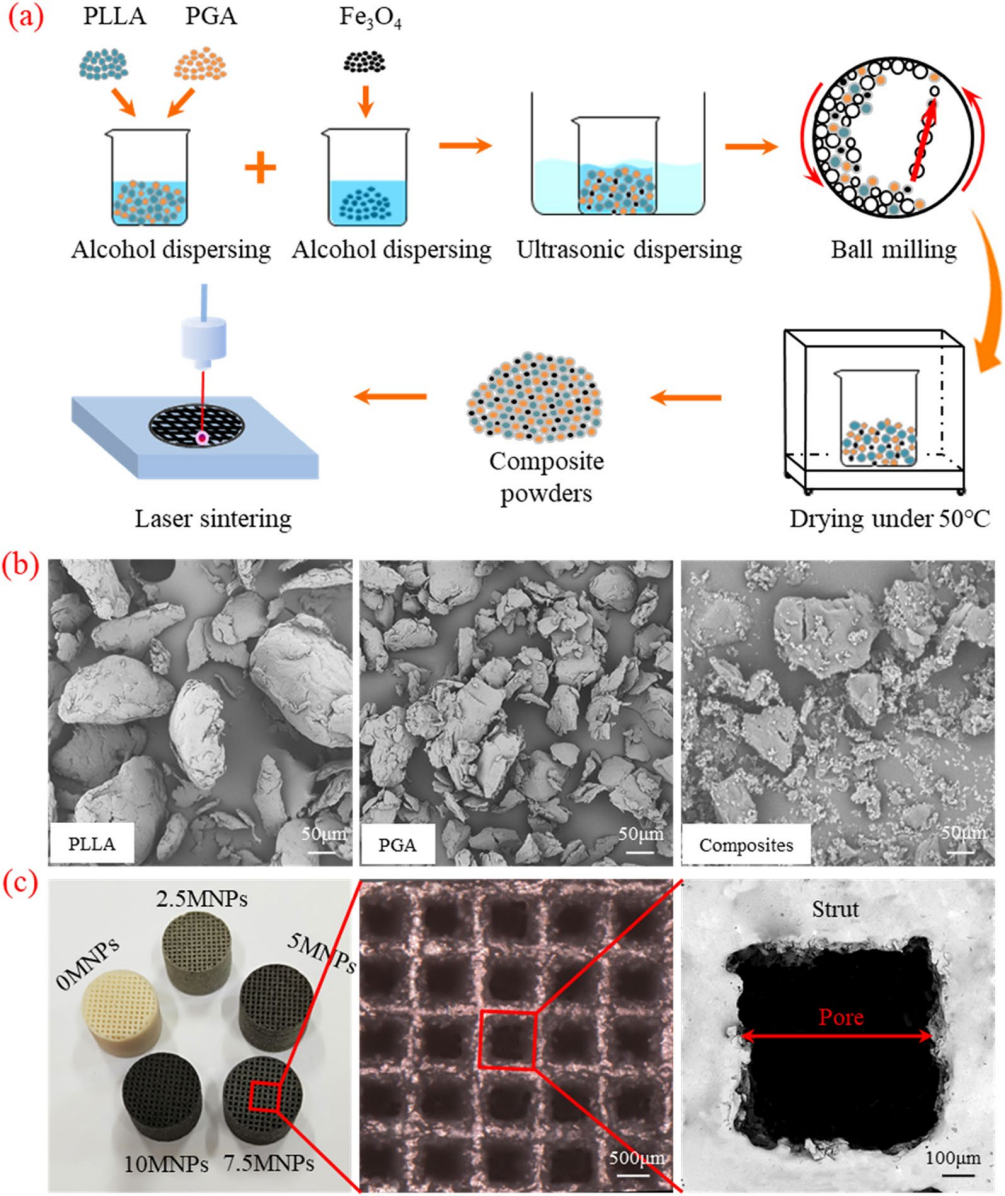
**a** Schematic illustration of PLLA/PGA/MNPs scaffolds preparation by selective laser sintering. **b** Powders of PLLA, PGA and composite. **c** PLLA/PGA scaffolds fabrication with different content of MNPs. Reprinted from Ref. [116]. Shuai, C., et al., A magnetic micro-environment in scaffolds for stimulating bone regeneration. Materials & Design, 2020. 185: p. 108275, open access article distributed under the CC BY-NC-ND license

On the other hand, the use of selective laser sintering for the fabrication of PLLA/PGA (polyglycolic acid) scaffolds containing MNPs Fe_3_O_4_ leads to the construction of magnetic scaffolds that provide cell adhesion and viability (Fig. 3). According to these studies, the stiffness of MNPs increased the modulus and compressive strength of the scaffold by 71.6% and 81.9%, and at the same time, it improved cell proliferation and alkaline phosphatase activity [116].

However, their validity and translation to the clinic require their functional evaluation in preclinical animal models, including mice, rats, and rabbits. Hence, in vivo imaging and ex vivo histological analysis is performed to monitor tissue development and measure them at the molecular level [141, 142]. After in vitro approval, the fabricated scaffolds were implanted in orthotopic and ectopic models, and collagen deposition, matrix mineralization, and bone tissue remodeling were investigated [124].

**Fig. 4:**
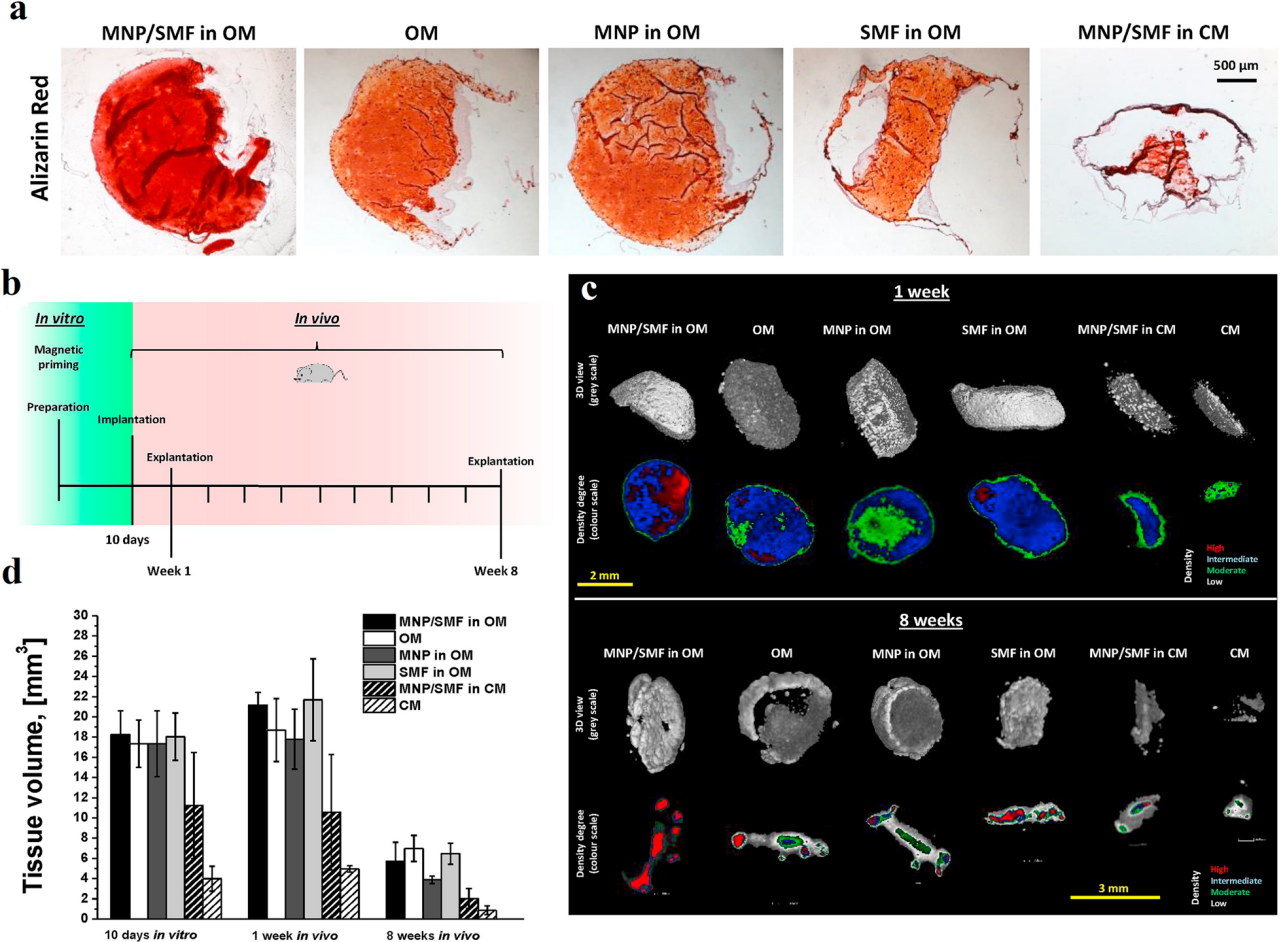
Osteogenic differentiation and bone formation. **a** After 3 weeks of culture, gel sections' mineralization (calcium deposition) was assessed using Alizarin red staining. **b** The samples were subcutaneously implanted in vivo, then observed via micro-computed tomography imaging after 1 or 8 weeks after extraction. **c** In vivo μCT image of gels in 3D volumetric view, and sections with gradient density of the tissue generated after 1 and 8 weeks. **d** By setting the following thresholds on the image histogram, the pixel intensity was defined based on the intervals of tissue density values: 0 to17000 (low density, in gray), 17,000 to 27,000 (moderate density, in green), 27,000 to 39,000 (intermediate density, in blue), and 39,000 to 65,500 (high density, in red). μCT analysis was used to calculate the tissue volume of gels. Reprinted from Ref. [143]. Filippi, M., et al., Magnetic nanocomposite hydrogels and static magnetic field stimulate the osteoblastic and vasculogenic profile of adipose-derived cells. Biomaterials, 2019. 223: p. 119468, Copyright (2023), with permission from Elsevier

As shown in Fig. 4, implantation of PEG-hydrogels-MNPs loaded with SVF (stromal vascular fraction) cells in nude mice indicated the formation of tissue with dense vascularization and high mineralization [143]. These results confirm that MNPs provide both matrix calcification and proliferation of endothelial cell in vitro and in vivo to form compact bone tissue in an implanted model. Furthermore, mesh of PCL polymer modified with Fe_3_O_4_ (15%) can stimulate vascularization and bone regeneration after implantation in segmental bone defects in mouse models [113]. The μCT imaging exposed that magnetic nanofibrous scaffolds and external SMF increased the formation of fresh homogeneous tissue in a rabbit within three months. The magnetic properties accelerate the remodeling of bone via the thorough absorption of the scaffold materials in damaged area [65].

**Fig. 5:**
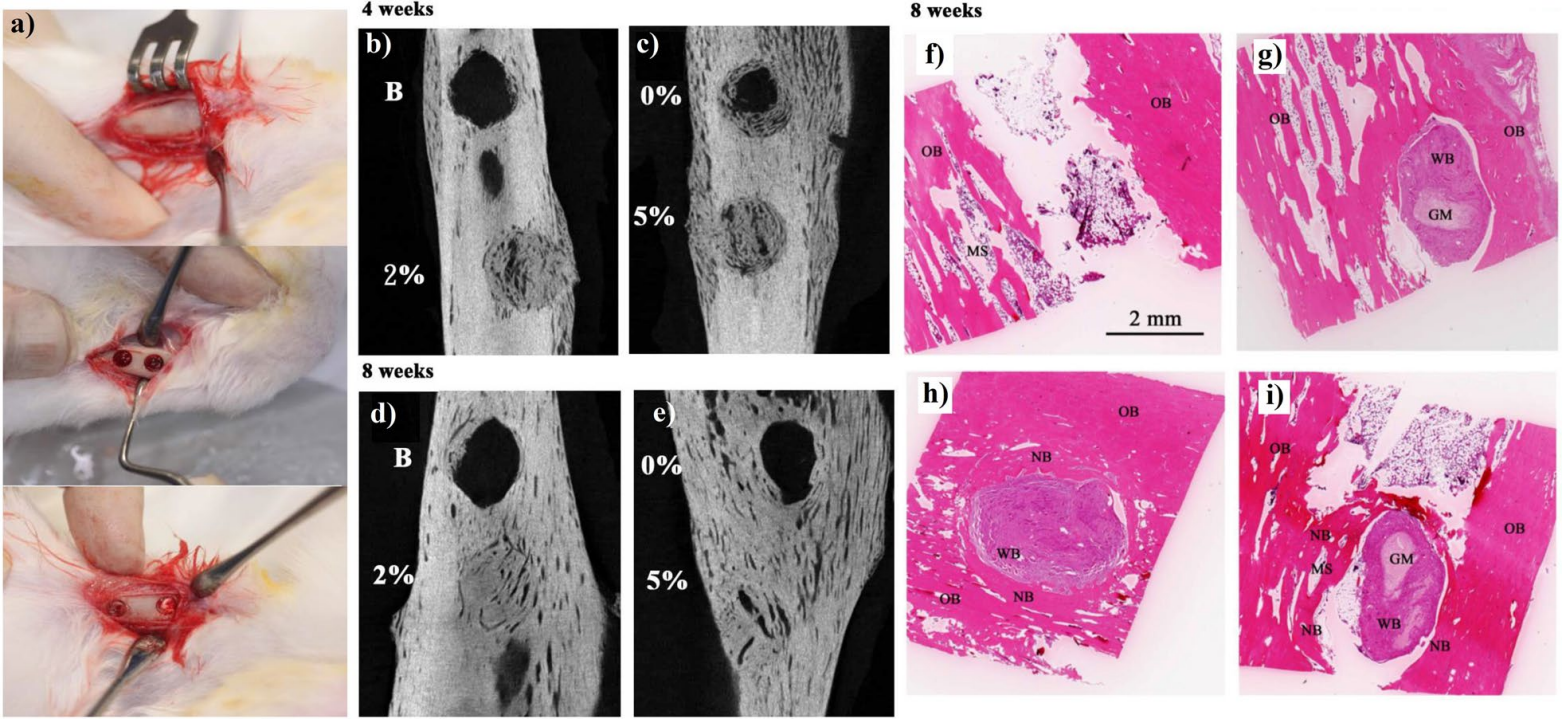
**a** Rabbit’s tibia was scraped and two implantation holes were prepared with a diameter of 4 mm. Next, neat PLLA was grafted into one defected site and PLLA/MNP was grafted into the other. **b**, **c** Micro-Computed Tomography (μCT) images of defect sites after 4 weeks and **d**, **e** 8 weeks. Histological images of defected sites grafted with: **f** blank; **g** PLLA; **h** 2% MNP/PLLA; and **i** 5% MNP/PLLA scaffolds after 8 weeks. Reprinted from Ref. [146]. Lai, W.-Y., et al., In vivo investigation into effectiveness of Fe_3_O_4_/PLLA nanofibers for bone tissue engineering applications. Polymers, 2018. 10(7): p. 804, open access article distributed under the terms and conditions of the Creative Commons Attribution (CC BY) license

In another study, four weeks of monitoring with MRI imaging revealed that implantation of SPION-enriched gelatin sponges in the Sprague–Dawley rats (incisor sockets) induced new bone formation and preserved alveolar [144]. In addition, CT imaging showed that the biodegradable Fe_3_O_4_ NPs/PLLA scaffolds accelerated the bone healing process after 8 weeks of implanting in tibia of rabbits [145]. Indeed, Fe_3_O_4_/PLLA nanofibers promoted bone regeneration in a dose-dependent behavior of nanoparticles, such that bone formation improved with increasing Fe_3_O_4_ NPs dosage [146] (Fig. 5).

**Fig. 6:**
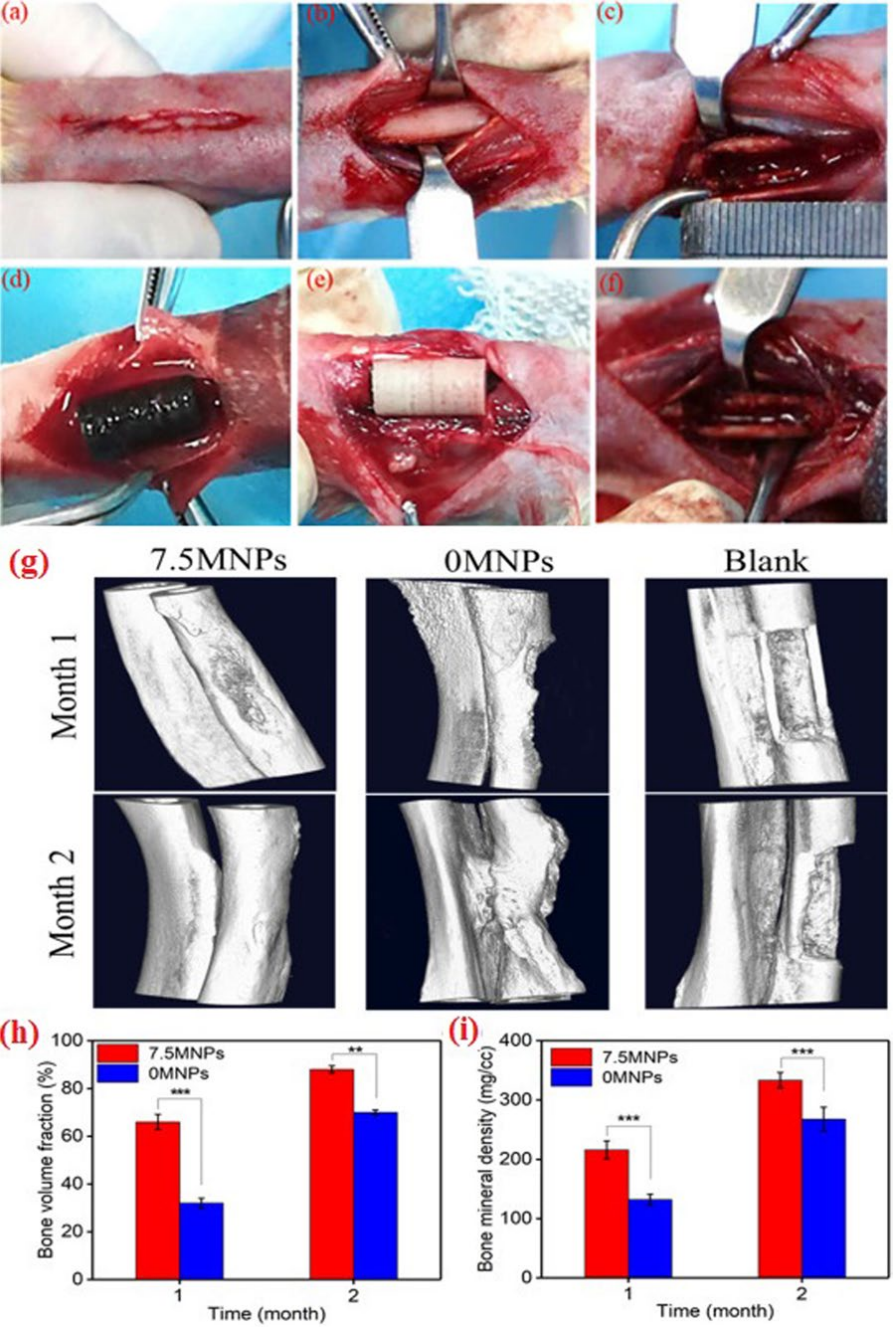
**a**–**c** The procedure involves the creation of a bone defect model. **d** Implantation of 7.5 MNPs scaffolds in rabbit radius defect site, **e** MNP-free scaffolds, and **f** defect site with no implantation. **g** Micro-CT images of bone defects treated with 7.5 MNPs scaffolds, 0 MNP scaffolds, and blank groups. **h** The bone volume fraction and **i** the bone mineral density in the 7.5 MNPs and 0 MNPs scaffold groups in 1 and 2 months after implantation (**P < 0.01 and ***P < 0.005). Reprinted from Ref. [116]. Shuai, C., et al., A magnetic micro-environment in scaffolds for stimulating bone regeneration. Materials & Design, 2020. 185: p. 108275, open access article distributed under the CC BY-NC-ND license

In another study, Shuai et al. prepared magnetic scaffolds and implanted in rabbit model within radius defect (Fig. 6). The results demonstrated that these structures remarkably increased angiogenesis, fibrous formation, and the creation of new bone tissue after two months [116]. Overall, the magnetic microenvironment may serve as an appropriate and efficient substrate in BTE in the future.

In general, MNPs and their nanocomposite scaffolds have several advantages over more conventional implants in bone tissue engineering **(**Table 1**)**. One advantage is that they can be functionalized with ligands that can target specific cells or tissues [147]. This allows for precise control over their localization and function, which is important for promoting tissue regeneration and minimizing unwanted side effects. Another advantage is the ability to manipulate magnetic nanoparticles through external magnetic fields, providing a non-invasive method for stimulating cells and promoting tissue regeneration, which is particularly important for bone tissue engineering [148]. MNPs can also modulate signaling pathways involved in bone formation and remodeling, such as the Wnt/β-catenin pathway, by functionalizing them with Wnt ligands or inhibitors, it is possible to promote or inhibit osteogenic differentiation, respectively, and regulate bone formation and remodeling [149]. Despite these advantages, there are several challenges that must be addressed before MNPs can be translated into clinical use. One important challenge is ensuring the long-term safety and biocompatibility of these materials. Magnetic nanoparticles have the potential to accumulate in tissues and organs over time, which could lead to toxicity and other adverse effects [150]. Researchers must carefully evaluate the biocompatibility of magnetic nanoparticles and their degradation products, as well as their potential to induce inflammation or other immune responses. Another challenge is understanding the mechanisms by which magnetic nanoparticles affect cell behavior and tissue regeneration. While there is growing evidence that magnetic nanoparticles can modulate signaling pathways involved in bone formation and remodeling, the underlying mechanisms are not yet fully understood. Further research is needed to elucidate the molecular and cellular mechanisms by which magnetic nanoparticles exert their effects, as well as their potential to interact with other signaling pathways and affect tissue regeneration in vivo.

## Conclusions

There are many studies on the application of MNPs alone or in the structure of nanocomposite scaffolds in TERM. A lot of these studies have also investigated and confirmed the physical and biological effects of MNPs in the microenvironment of stem cells. In particular, the increasing current advanced research in BTE and growing amounts of experimental data point to the high potential of MNPs in the repair and healing of bone injuries. According to the conducted studies, incorporation and homogeneous distribution of MNPs in scaffolds leads to biological effects on cell activity and plays a role in their fate toward osteogenic differentiation. In this regard, many researchers investigated the molecular mechanisms and signaling pathways related to the osteogenic differentiation of stem cells and related or helpful factors in bone healing, especially angiogenesis, in the presence of MNPs or scaffolds containing them. Finally, the activation of molecular mechanisms and signaling pathways related to bone regeneration, as well as the creation of angiogenic responses and the formation of blood vessels and capillary tubes have been confirmed in recent years. Therefore, according to the present review, scaffolds reinforced with MNPs have been very helpful and effective in the reconstruction and healing of damaged bones. It is expected that in the near future, great achievements will be made available in the field of bone reconstruction using magnetic nanocomposite products. To achieve this important goal in bone tissue engineering, biosafety issues in the field of magnetization strategies should be further investigated. Although so far in vivo studies of magnetic scaffolds have not shown any serious toxic and inflammatory effects, these studies need to be completely conducted in vivo and over a longer period of time.

## Data Availability

Not applicable**.**

## Abbreviations

MNPs: Magnetic nanoparticles
MSCs: Mesenchymal stem cells
BTE: Bone tissue engineering
EMFs: Electromagnetic fields
SMFs: Static magnetic fields
SPIONs: Superparamagnetic iron oxide nanoparticles
MRI: Magnetic resonance imaging
ECM: Extracellular matrix
FDA: Food and Drug Administration
MRS: Magnetic responsive scaffolds
ALP: Alkaline phosphatase
PLLA: Poly (l-lactic acid)
CPC: Calcium phosphate cement
hDPSCs: Human dental pulp stem cells
HA: Hydroxyapatite
CZF: Cobalt-zinc ferrite
PCL: Poly (ε-caprolactone)
h-BMSCs: Human bone marrow mesenchymal stem cells
PVDF: Poly (vinylidene fluoride)
PGA: Poly (glycolic acid)
SVF: Stromal vascular fraction
BMP2: Bone morphogenetic protein 2
MAPK: Classic mitogen-activated protein kinase
INZEB_2_: Long non-coding RNA
BV/TV: Bone volume per tissue volume
TEM: Transmission electron microscope
IHC: Immunohistochemistry
Runx2: Runt-related transcription factor 2
GSK3: Glycogen synthase kinase-3
GO: Graphene oxide
PEMF: Pulsed electromagnetic field
PKA: Protein kinase A
JNK: C-Jun N-terminal kinase
VEGF: Vascular endothelial growth factor
